# Supplementary material for: A Humanized Yeast Phenomic Model of Deoxycytidine Kinase to Predict Genetic Buffering of Nucleoside Analog Cytotoxicity
Source: Genes (Basel). 2019 Sep 30;10(10):770. doi: 10.3390/genes10100770 (PMC6826991; doi:10.3390/genes10100770)
Supplement: Supplementary file 1 [file genes-10-00770-s001.zip › Additional_Files/Additional_File7_GOTermHeatmaps.zip!/Additional_File7_GOTermHeatmaps/DNA_replication_initiation.pdf]

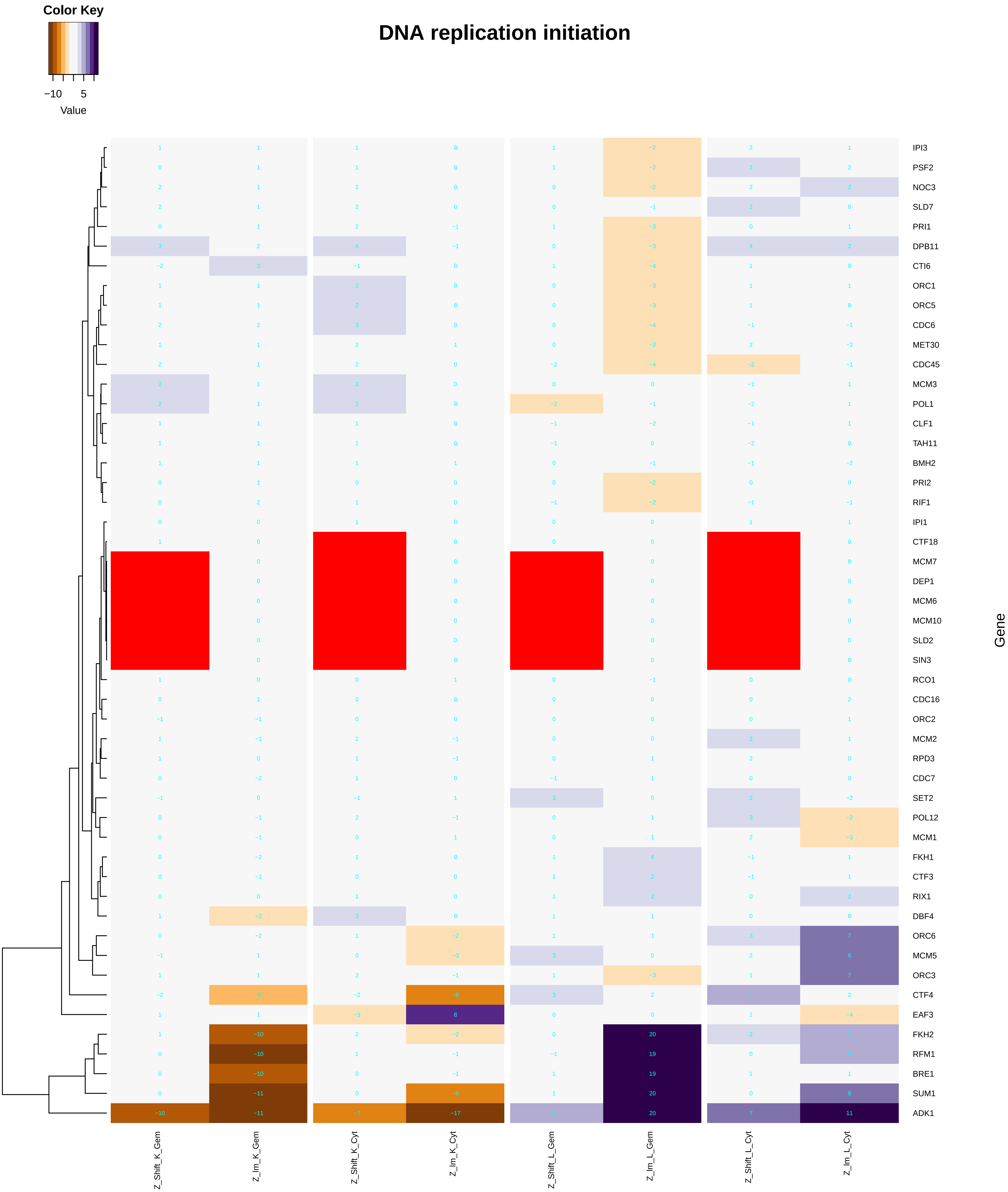

DNA replication initiation
Color Key
−10
5
Value
IPI3
PSF2
NOC3
SLD7
PRI1
DPB11
CTI6
ORC1
ORC5
CDC6
MET30
CDC45
MCM3
POL1
CLF1
TAH11
BMH2
PRI2
RIF1
IPI1
CTF18
MCM7
DEP1
MCM6
MCM10
SLD2
SIN3
RCO1
CDC16
ORC2
MCM2
RPD3
CDC7
SET2
POL12
MCM1
FKH1
CTF3
RIX1
DBF4
ORC6
MCM5
ORC3
CTF4
EAF3
FKH2
RFM1
BRE1
SUM1
ADK1
Gene
Z_Shift_K_Gem
Z_Im_K_Gem
Z_Shift_K_Cyt
Z_Im_K_Cyt
Z_Shift_L_Gem
Z_Im_L_Gem
Z_Shift_L_Cyt
Z_Im_L_Cyt

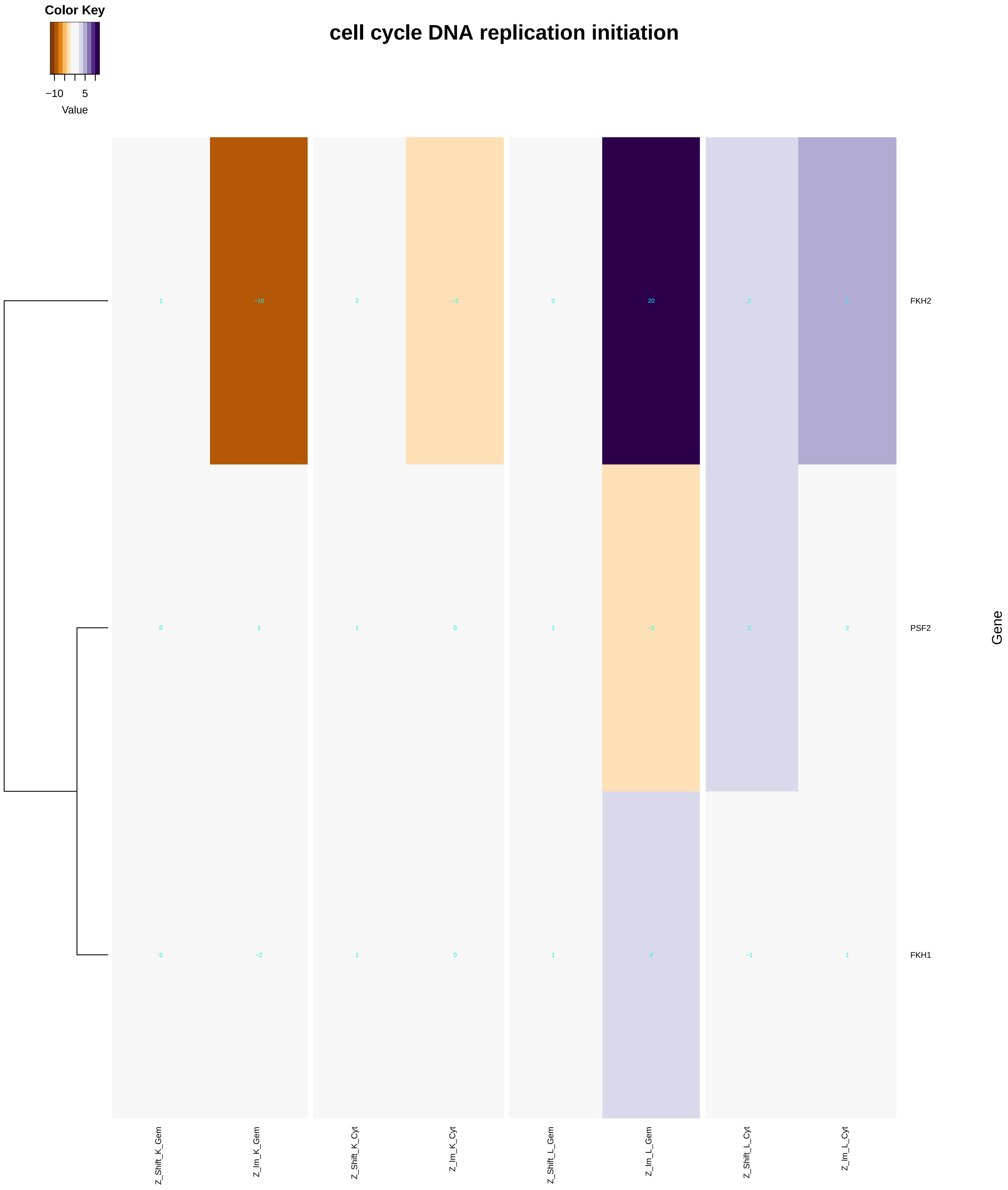
Color Key
−10
5
Value
cell cycle DNA replication initiation
1
−10
2
−2
0
20
2
5
FKH2
0
1
1
0
1
−2
2
2
PSF2
0
−2
1
0
1
4
−1
1
FKH1
Gene
Z_Shift_K_Gem
Z_Im_K_Gem
Z_Shift_K_Cyt
Z_Im_K_Cyt
Z_Shift_L_Gem
Z_Im_L_Gem
Z_Shift_L_Cyt
Z_Im_L_Cyt

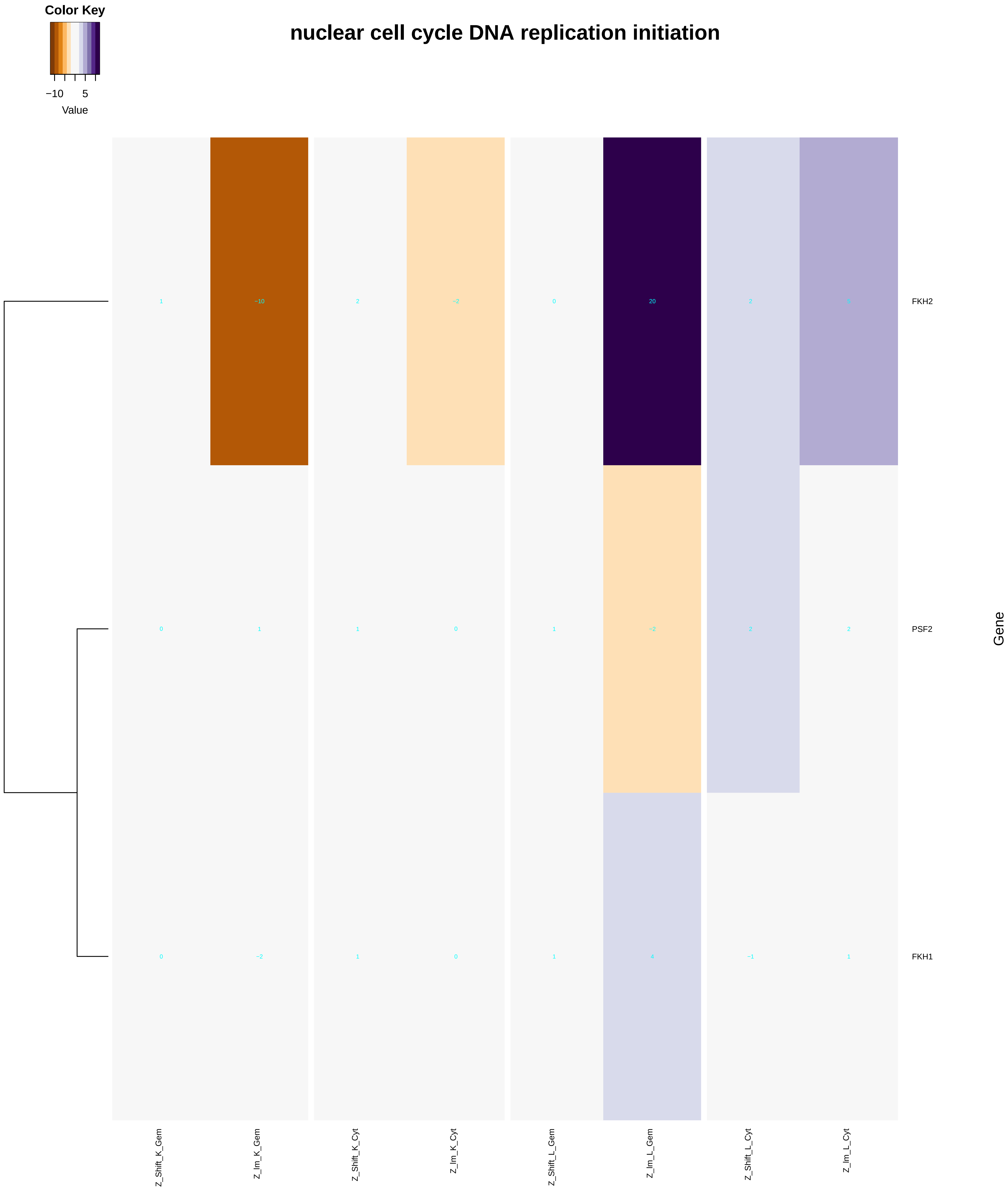
Color Key
−10
5
Value
nuclear cell cycle DNA replication initiation
1
−10
2
−2
0
20
2
5
FKH2
0
1
1
0
1
−2
2
2
PSF2
0
−2
1
0
1
4
−1
1
FKH1
Gene
Z_Shift_K_Gem
Z_Im_K_Gem
Z_Shift_K_Cyt
Z_Im_K_Cyt
Z_Shift_L_Gem
Z_Im_L_Gem
Z_Shift_L_Cyt
Z_Im_L_Cyt

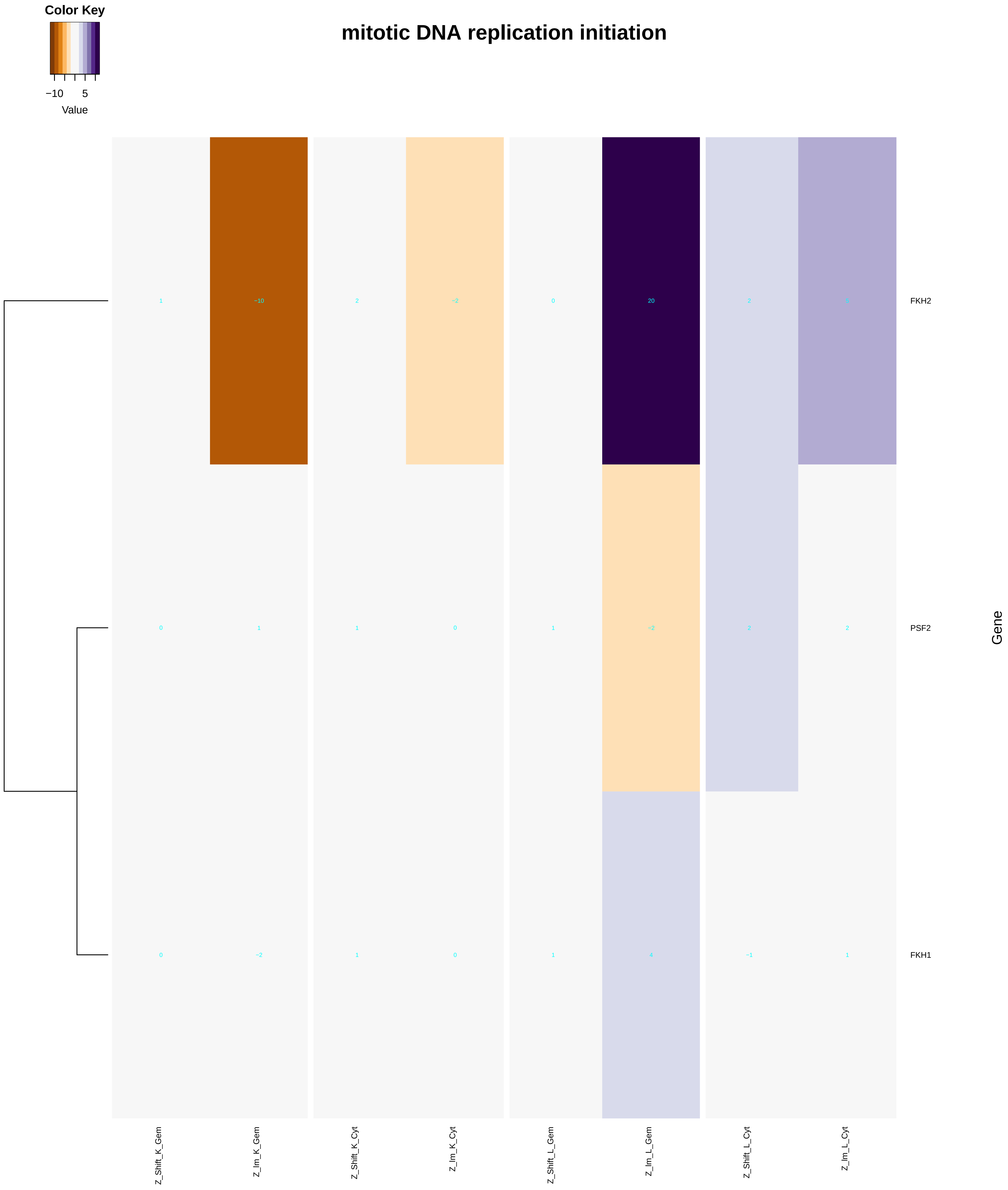
Color Key
−10
5
Value
mitotic DNA replication initiation
1
−10
2
−2
0
20
2
5
FKH2
0
1
1
0
1
−2
2
2
PSF2
0
−2
1
0
1
4
−1
1
FKH1
Gene
Z_Shift_K_Gem
Z_Im_K_Gem
Z_Shift_K_Cyt
Z_Im_K_Cyt
Z_Shift_L_Gem
Z_Im_L_Gem
Z_Shift_L_Cyt
Z_Im_L_Cyt
